# Diversity In Cortical Thymic Epithelial Cells Occurs Through Loss Of A Foxn1-Dependent Gene Signature Driven By Stage-Specific Thymocyte Crosstalk^1^

**DOI:** 10.4049/jimmunol.2200609

**Published:** 2022-11-14

**Authors:** Andrea J. White, Sonia M. Parnell, Adam Handel, Stefano Maio, Andrea Bacon, Emilie J. Cosway, Beth Lucas, Kieran D. James, Jennifer E. Cowan, William E. Jenkinson, Georg A. Hollander, Graham Anderson

**Affiliations:** * Institute of Immunology and Immunotherapy, University of Birmingham, Birmingham, UK; ‡ Department of Paediatrics and Institute of Developmental and Regenerative Medicine, University of Oxford, Oxford, UK; ¶ Nuffield Department of Clinical Neurosciences, University of Oxford, Oxford, UK; § Paediatric Immunology, Department of Biomedicine, University of Basel and University Children’s Hospital Basel, Basel, Switzerland; # Department of Biosystems Science and Engineering, ETH Zurich, Basel, Switzerland; † Division of Infection and Immunity, University College London, London, UK

## Abstract

In the thymus, cortical and medullary thymic epithelial cells (TEC) support αβT-cell development from lymphoid progenitors. For cortical TEC (cTEC), expression of a specialised gene signature that includes *Cxcl12*, Delta-like 4 (*Dll4*) and *Psmb11* enables the cortex to support T-lineage commitment and the generation and selection of CD4^+^CD8^+^ thymocytes. While the importance of cTEC in T-cell development is well defined, mechanisms that shape the cTEC compartment and regulate its functional specialisation are unclear. Using a *Cxcl12*^DsRed^ reporter mouse model, we show that changes in *Cxcl12* expression reveal a developmentally regulated programme of cTEC heterogeneity. While cTEC are uniformly *Cxcl12*^DsRed+^ during neonatal stages, progression through postnatal life triggers the appearance of *Cxcl12*^DsRed-^ cTEC that continue to reside in the cortex alongside their *Cxcl12*^DsRed+^ counterparts. This appearance of *Cxcl12*^DsRed-^ cTEC is controlled by maturation of CD4^-^CD8^-^, but not CD4^+^CD8^+^ thymocytes, demonstrating stage-specific thymocyte crosstalk controls cTEC heterogeneity. Importantly, while fate mapping experiments show both *Cxcl12*^DsRed+^ and *Cxcl12*^DsRed-^ cTEC share a common *Foxn1*^+^ cell origin, RNA sequencing analysis shows *Cxcl12*^DsRed-^ cTEC no longer express *Foxn1*, which results in loss of the FOXN1-dependent cTEC gene signature and may explain the reduced capacity of *Cxcl12*^DsRed-^ cTEC for thymocyte interactions. In sum, our study shows that shaping of the cTEC compartment during the life-course occurs via stage-specific thymocyte crosstalk, which drives loss of *Foxn1* expression and its key target genes which may then determine the functional competence of the thymic cortex.

## Introduction

Self-tolerant MHC restricted CD4^+^ and CD8^+^ αβT-cells are produced exclusively in the thymus, a primary lymphoid organ that guides lymphoid progenitors through multiple developmental events. Importantly, many studies have shown the key roles that thymic stromal cells play in controlling thymocyte development (1–3). In particular, thymic epithelial cells (TEC) are functionally important during multiple developmental events that occur within anatomically distinct thymic areas(4). For example, EpCAM1^+^UEA1^+^Ly51^-^ medullary thymic epithelial cells (mTEC) are key in controlling T-cell tolerance induction through the induction of both negative selection and Foxp3^+^ T-cell development (5, 6). In contrast, cortex-resident cortical TEC (cTEC), typically defined as EpCAM1^+^ UEA1^-^ Ly51^+^ cells, are critical regulators of early T-cell development. For example, upon entry to the thymus, lymphoid progenitors undergo interactions with Delta-like 4 (DLL4) expressing cTEC, which induces Notch signalling and directs progenitors towards a T-cell fate (7–9). Immature thymocytes then transit through a series of CD4^-^CD8^-^ double negative (DN) stages, including CD44^+^CD25^-^ DN1, CD44^+^CD25^+^ DN2 and CD44^-^CD25^+^ DN3, where they rearrange the *Tcrb* gene and express TCRβ protein as part of the cell surface pre-TCR complex. Importantly, selection of TCRβ-expressing DN3 cells is also controlled by cTEC products, with CXCL12 and DLL4 acting in concert with the pre-TCR to generate large cohorts of pre-selection CD4^+^CD8^+^αβTCR^low^ thymocytes (10, 11). cTEC expression of MHC/self-peptide complexes then enables the cortex to support positive selection of CD4^+^CD8^+^ thymocytes, that results in the generation of single positive (SP) CD4^+^ and CD8^+^ thymocytes. Here, the unique ability of cTEC to support positive selection is at least in part attributed to their specialised antigen processing capabilities (12). For example, unique expression of *Psmb11*, the gene encoding the thymoproteosomal subunit β5t, enables cTEC to produce MHC class I bound self-peptides that result in the effective positive selection of CD8^+^ thymocytes (13, 14). Similarly, cTEC expression of Cathepsin-L (15) and Prss16 (16) enables the generation MHC class II/self-peptide complexes that drive efficient CD4^+^ thymocyte selection. Autophagic properties of cTEC may also aid in their control of positive selection(17). Significantly, many of the genes expressed by cTEC that underpin their functional specialisation, including *Cxcl12*, *Dll4*, *Psmb11* and *Ctsl*, are known targets of FOXN1 (18, 19), a transcription factor that plays an essential role in TEC development and function (20–22). Thus, cTEC expression of FOXN1 plays an important role in controlling a key gene expression signature that enables the cortex to support multiple stages of T-cell development.

Despite this importance of cTEC for thymus function, our understanding of the mechanisms that control their development remains incomplete. To address cTEC development and heterogeneity, we examined Ly51^+^UEA1^-^ cTEC for evidence of heterogeneity using mice in which the fluorescent protein DsRed reports expression of the functionally important cTEC gene *Cxcl12* (23). We found that cTEC in adult mice can be readily subdivided into *Cxcl12*^DsRed+^ and *Cxcl12*^DsRed-^ subsets that both reside within the thymic cortex, with qPCR analysis confirming their differential *Cxcl12* gene expression. Interestingly, examination of cTEC heterogeneity across the life-course revealed a developmentally regulated programme where cTEC were uniformly *Cxcl12*^DsRed+^ at neonatal stages, with *Cxcl12*^DsRed-^ cTEC appearing 1 week after birth and persisting into adulthood. Importantly, while fate mapping experiments show *Cxcl12*^DsRed+^ and *Cxcl12*^DsRed-^ cTEC both derive from FOXN1^+^ cells, RNA sequencing analysis showed these populations to be transcriptionally distinct. Unlike *Cxcl12*^DsRed+^ cTEC, *Cxcl12*^DsRed-^ cTEC lacked *Foxn1* expression, and this was accompanied by a change in the gene expression profiles of FOXN1 targets, including *Cxcl12* itself, as well as *Psmbl11*, and the Notch ligand *Dll4*. Furthermore, the emergence of *Cxcl12*^DsRed-^ cTEC was impaired in *Rag2*^-/-^ but not *Tcra*^-/-^ mice, and *Cxcl12*^DsRed-^ cTEC were impaired in their ability to form successful cellular interactions with thymocytes when compared to their *Cxcl12*^DsRed+^ counterparts. Taken together, our study identifies a developmentally regulated programme of cTEC heterogeneity, where signals arising from the maturation of immature DN3 thymocytes cause transcriptional changes in the cTEC population that result in loss of *Foxn1* expression and transcripts of its downstream targets. This then creates epithelial heterogeneity in the thymic cortex that may influence functionality within the cTEC compartment.

## Materials and Methods

### Mice

The following mice on a C57BL/6 background were purchased from The Jackson Laboratory and used at 10 weeks of age unless otherwise stated: *Cxcl12*^DsRed^ knockin (stock no. 022458) (23), which were used in isolation or crossed with the following: *Tcr*α^-/-^ (stock no. 002116, (24), *Rag2*^-/-^(stock no. 008449) (25), *Foxn1*^Cre^ (stock no. 018448)(26) and Rosa26-stop-EYFP (stock no. 006148) (27). Control mice for experiments involving and *Tcra*^-/-^ and *Rag2*^-/-^ mice were heterozygous littermate controls. RANK^Venus^ BAC transgenic mice were generated as described previously (28). Husbandry, housing, and experimental methods involving mice were performed at the Biomedical Services Unit at the University of Birmingham in accordance with the local Ethical Review Panel and UK Home Office Regulations (Animal project Licence number P3ACFED06).

### Flow Cytometry, Cell Sorting and Antibodies

For thymic epithelial cell analysis, single cell suspensions were generated by digesting thymic lobes with collagenase dispase (2.5mg/ml, Roche) and DNase 1 (40mg/ml Roche). CD45^-^ cells were enriched by the depletion of CD45^+^ cells using anti-CD45 beads and LS columns (Miltenyi Biotec). The following antibodies were used for TEC analysis: anti-CD45 clone 30-F11 (eBioscience), anti-EpCAM1 clone G8.8 (eBioscience), anti-Ly51 clone 6C3 (Biolegend), anti-MHCII clone M5/114.15.2 (eBioscience), anti-CD80 clone 16-10A1 (Biolegend), CD104 clone 346-11A (Biolegend), and anti-MHCI 28-14-8. Biotinylated UEA-1 (Vector laboratories) was detected using streptavidin PECy7 (eBioscience). Cells were analysed using a LSR Fortessa (Becton Dickinson) with data analysis carried out using Flowjo v10 (Becton Dickinson). For cell sorting, TEC subsets were identified using the antibodies above, and isolated using a FACS Aria Fusion 1 cell sorter (Becton Dickinson).

The sorting strategy for the different TEC subsets were as follows, Cxcl12^DsRed+^ cTEC: CD45^-^EpCAM1^+^UEA1^-^Ly51^+^CXCL12^DsRed+^; CXCL12^DsRed-^ cTEC: CD45^-^EpCAM1^+^UEA1^-^Ly51^+^CXCL12^DsRed-^; mTEC^lo^: CD45^-^EpCAM1^+^UEA1^+^Ly51^-^CD80^-^MHCII^-^; mTEC^hi^: CD45^-^EpCAM1^+^UEA-1^-^Ly51^+^CD80^+^MHCII^+^; CD104^+^ mTEC^lo^, CD45^-^EpCAM1^+^UEA1^+^Ly51^-^CD80^-^MHCII^-^CD104^+^; CD104^-^ mTEC^lo^, CD45^-^EpCAM1^+^UEA1^+^Ly51^-^CD80^-^MHCII^-^CD104^-^.

### Immunohistochemistry and Confocal Microscropy

Thymus tissue from *Foxn1*^Cre^/*Rosa26*^YFP^/*Cxcl12* mice was isolated and fixed in 2% PFA (Sigma) for 2 hours, then overnight in 15% Sucrose (Sigma). Thymic lobes were frozen on dry ice, and sectioned at 7μm within 24 hours of freezing. eYFP protein in sections from *Foxn1*^Cre^/*Rosa26*^YFP^/*Cxcl12*^DsRed^ was amplified using rabbit anti-GFP (ThermoFisher) and donkey anti-rabbit 488 (ThermoFisher). Sections were counter stained with DAPI (4’,6-diamidino-2-phenylindole)(Sigma) and mounted using Prolong Diamond (ThermoFisher). Sections were imaged using Zeiss Zen 880 microscope (Zeiss) and analysis using Zeiss Zen Black (Zeiss).

### qPCR Analysis

Real-time PCR was performed as described previously(29) on a Corbett Rotor Gene-3000 PCR machine (Qiagen) using a SensiMix SYBR No ROX Kit (Meridian Bioscience-Bioline) and primers specific for *Actb* (β-actin) (Qiagen), and indicated genes of interest (Sigma-Merck). Data shown are typical of at least two independently sorted sample sets, histograms represent the mean (±SEM) of replicate reactions. Primer sequences used were:

*Foxn1*: forward 5′-*CAAATTCTGCAGGGGTCAGA*-3′ and reverse 5′-*TGGGGTGCAATCCTCTGATA*-3′;

*Cxcl12*: forward 5′-*GCTCTGCATCAGTGACGGTA*-3′ and reverse 5′-*TGTCTGTTGTTGTTCTTCAGC*-3′;

*Psmb11*: forward 5′-*ATCGCTGCGGCTGATACTC*-3′ and reverse 5′-*GCAGGACATCATAGCTGCCAA*-3′;

*Prss16*: forward 5′-*GTATTTCTGCACATAGGAGGCG*-3′ and reverse 5′-*TGTTCTAGGCTTATCACCAGGG*-3′;

*Cd83*: forward 5′-*AGGGCCTATTCCCTGACGAT*-3′ and reverse 5′-*CTTCCTTGGGGCATCCTGTC*-3′;

*Dll4*: forward 5′-*GAAGCGCGATGACCACTTCG*-3′ and reverse 5′-*TGGACGGCAGATGCACTCAT*-3′;

*Ly75*: forward 5′-*GCTCAGGTAATGATCCATTCACC*-3′ and reverse 5′-*TTAGTTCCGCTACAGTCCTGG*-3′;

*Ctsl*: forward 5′-*ATCAAACCTTTAGTGCAGAGTGG*-3′ and reverse 5 ′-*CTGTATTCCCCGTTGTGTAGC*-3′;

*Epcam1*: forward 5′-*TTGCTCCAAACTGGCGTCTAA*-3′ and reverse 5′-*GCAGTCGGGGTCGTACA*-3′;

*Aire*: forward 5′-*TGCATAGCATCCTGGACGGCTTCC*-3′ and reverse 5′-*CCTGGGCTGGAGACGCTCTTTGAG*-3′;

*Trpm5*: forward 5′-*CCAGCATAAGCGACAACATCT*-3′ and reverse 5′-*GAGCATACAGTAGTTGGCCTG*-3′;

*Ccl21a*: forward 5′-*ATCCCGGCAATCCTGTTCTC*-3′ and reverse 5′-*GGGGCTTTGTTTCCCTGGG*-3′;

*Actb* (β-actin): QuantiTect Mm *Actb* 1SG Primer Assay (Qiagen, QT00095242).

### Bulk RNA Sequencing

RNA samples were extracted using the QIagen RNAeasy kit. Libraries were prepared using the SMARTer Ultra Low Input RNA Kit for Sequencing as per manufacturer instructions and sequenced on an Illumina NovaSeq platform. Reads were trimmed for adapter contamination using Trimmomatic (version 0.36) and aligned to the mm10 mouse genome using STAR (version 2.7.3a) (30, 31). Reads were assigned to genes using HTSeq (version 0.12.4) with the option “intersection-nonempty” (32). Differentially expressed genes were identified using edgeR (FDR < 0.05) (33). Enrichment of *Foxn1* high confidence genes (18) was assessed by comparing the log_2_ fold expression for *Foxn1* high confidence genes to a control set of genes matched by expression decile using a Wilcoxon rank sum test. Sequencing data is available at the Gene Expression Omnibus GEO (https://www.ncbi.nlm.nih.gov/geo/query/acc.cgi?acc=GSE205940. Accession number GSE205940). Gene ontology analysis was performed using clusterProfiler (34).

### Cell Conjugate Analysis

Thymocyte-TEC cell conjugate experiments were carried out using protocol adapted from Hare et al (35). In short, CD45^-^EpCAM1^+^ TEC were FACS-sorted from 10 week *Cxcl12*^DsRed^ mice and neonatal day 2 wildtype mice, and labelled with CFSE according to manufacturer instructions (ThermoFisher). A single cell suspension of WT adult thymocytes were labelled with Cell Trace Violet according to manufacturer’s instructions (ThermoFisher), and the two cell types were mixed at a 5:1 ratio (Thymocytes:TEC). The mixed suspension was then centrifuged, the supernatant removed, and the cell pellet vortexed and incubated at 37°C for 20 minutes, a timepoint that enables successful conjugate formation between WT TEC and thymocytes (35). Samples were resuspended in a volume of 200μl of PBS (Sigma), and analysed using a BD LSR Fortessa.

## Results

### Progressive Loss of *Cxcl12* Expression Identifies A Developmentally Regulated Programme of cTEC Heterogeneity

**Figure 1:**
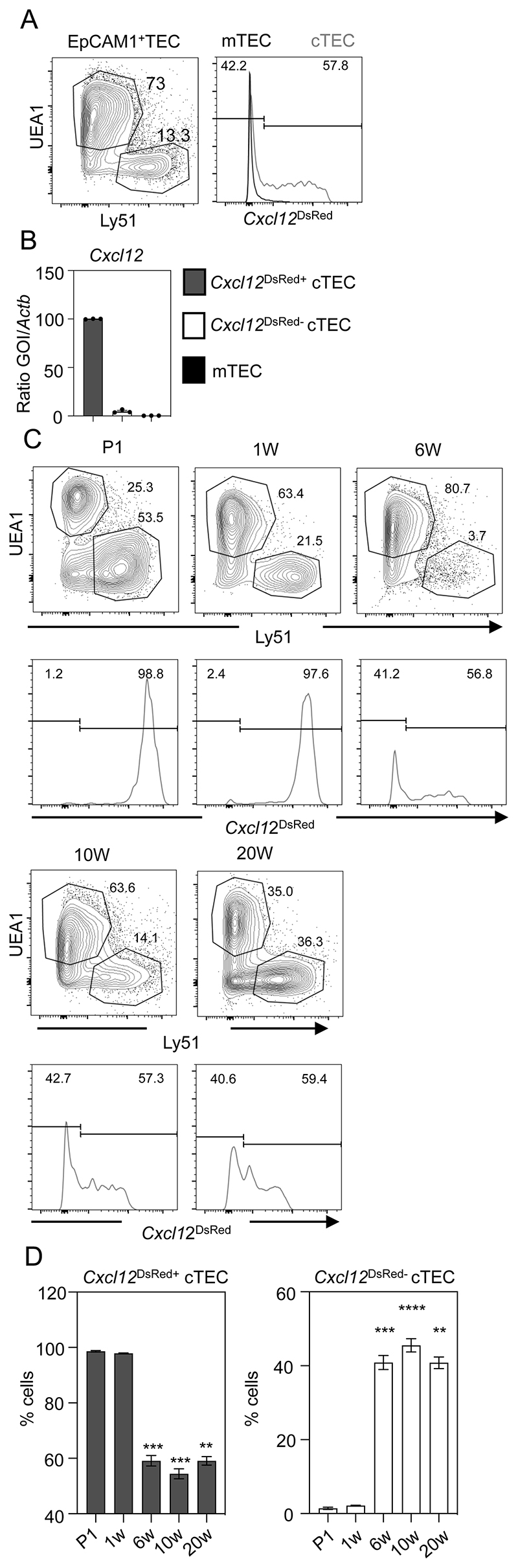
*Cxcl12* Expression Defines Developmentally Controlled Heterogeneity in cTEC. (A) Flow cytometric analysis of EpCAM1^+^CD45^-^ thymic epithelial cells (TEC) from adult 10 week *Cxcl1*2^DsRed^ mice, separated into UEA1^+^Ly51^-^ mTEC and UEA1^-^Ly51^+^ cTEC. Levels of *Cxcl12*^DsRed^ expression in cTEC (gray line) and mTEC (black line) are shown. (B) shows qPCR expression of *Cxcl12* mRNA in FACS-sorted *Cxcl12*^DsRed+^ and *Cxcl12*^DsRed-^ cTEC subsets, with mTEC shown for comparison. (C) shows time-course analysis of *Cxcl12*^DsRed^ expression in cTEC, identified using the gating shown, as UEA1^-^Ly51^+^ cells, from mice at indicated ages. Gates are set using mTEC as in (A). (D) shows quantitation of *Cxcl12*^DsRed+^ and *Cxcl12*^DsRed-^ cTEC subsets. Each time point is from a minimum of n=4 mice and at least 3 separate experiments: P1 n=5, 1W n=4, 6W n=5, 10W n=9, and 20W n=6. P values are as follows and indicate the significance relative to P1, using a Mann-Whitney non-parametric test: **, P < 0.01; ***, P < 0.001; and ****, P < 0.0001. Error bars represent mean ± SEM.

In the thymus, cTEC are classically defined as an Ly51^+^UEA1^-^ subset of EpCAM1^+^ TEC. While the functional properties of cTEC are well described, relatively little is known about the cellular and molecular interactions that control their development and potential functional heterogeneity. To investigate this, we made use of *Cxcl12*^DsRed^ reporter mice(23) in which DsRed expression identifies cells expressing *Cxcl12*, a cTEC-expressed chemokine that is an important regulator of thymocyte migration and development. Surprisingly, flow cytometric analysis of Ly51^+^UEA1^-^ cTEC from 10 week old adult *Cxcl12*^DsRed^ mice revealed striking heterogeneity with regard to DsRed expression, with the presence of distinct subsets of *Cxcl12*^DsRed+^ and *Cxcl12*^DsRed-^ cTEC (Fig. 1A). Importantly, when FACS-purified DsRed^+^ and DsRed^-^ cTEC cells were analysed for *Cxcl12* mRNA expression by qPCR, we saw that the abundant expression of *Cxcl12* mRNA in DsRed^+^ cells was lacking in DsRed^-^ cells (Fig 1B). Thus, heterogeneity in adult cTEC described here reflects true heterogeneity in their *Cxcl12* expression, and is not merely a feature of DsRed reporter expression.

To examine cTEC heterogeneity further, we performed time-course analysis from birth up to 20 weeks of adulthood. Interestingly, we saw that cTEC from neonatal (postnatal day 1, P1) were uniformly *Cxcl12*^DsRed+^ (Fig. 1C). While the vast majority of cTEC were also Cxcl12^DsRed+^ at the 1 week stage, we detected a distinct *Cxcl12*^DsRed-^ cTEC subset at 6 weeks of life (Fig. 1C), with the proportions of *Cxcl12*^DsRed+^ and *Cxcl12*^DsRed-^ cTEC remaining constant for the remainder of the observation period (Fig. 1C, D). Collectively, these findings identify *Cxcl12*^+^ and *Cxcl12*^-^ subsets within the bulk cTEC compartment that are ordered in their appearance during development, suggesting the cTEC compartment undergoes developmentally regulated changes that can be measured by differences in *Cxcl12* expression.

### *Cxcl12*^DsRed-^ cTEC Are Transcriptionally Distinct from Their *Cxcl12*^DsRed+^ Counterparts, And Lack *Foxn1* Expression and a FOXN1-Dependent Gene Signature

**Figure 2:**
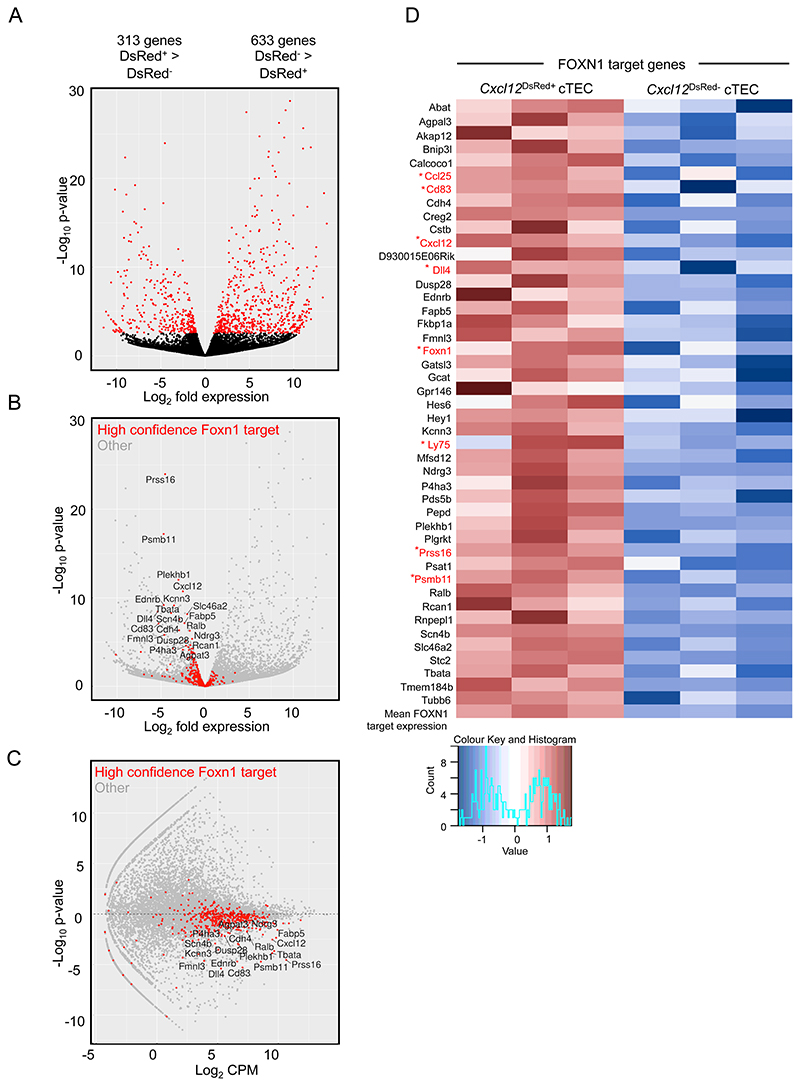
*Cxcl12*^DsRed+^ and *Cxcl12*^DsRed-^ cTEC Subsets Are Transcriptionally Distinct. RNA-seq analysis of FACS-sorted *Cxcl12*^DsRed+^ and *Cxc*l*12*^DsRed-^ cTEC from 10 week *Cxcl12^DsRed^* mice. (A) shows a volcano plot of differentially expressed genes between cTEC subsets, red dots represent FDR<0.05 and black dots represent no significance. (B) shows a volcano plot of differentially expressed genes, emphasising significant FOXN1 high confidence target genes shown by red dots, with all other genes represented by grey dots. (C) shows an MA plot for all genes highlighting high confidence FOXN1 target genes in red, other genes shown in grey. Graphs show a technical triplicate of a single experiment that is representative of 3 individally sorted biological replicates. (D) shows a heatmap of significantly differentially expressed FOXN1 target genes as identified in Zuklys et al (18), and scaled mean expression of all FOXN1 target genes. Only FOXN1 targets with mean expression >1 counts per million (CPM) were included. Genes associated with cTEC phenotype/function are highlighted in red.

**Figure 3:**
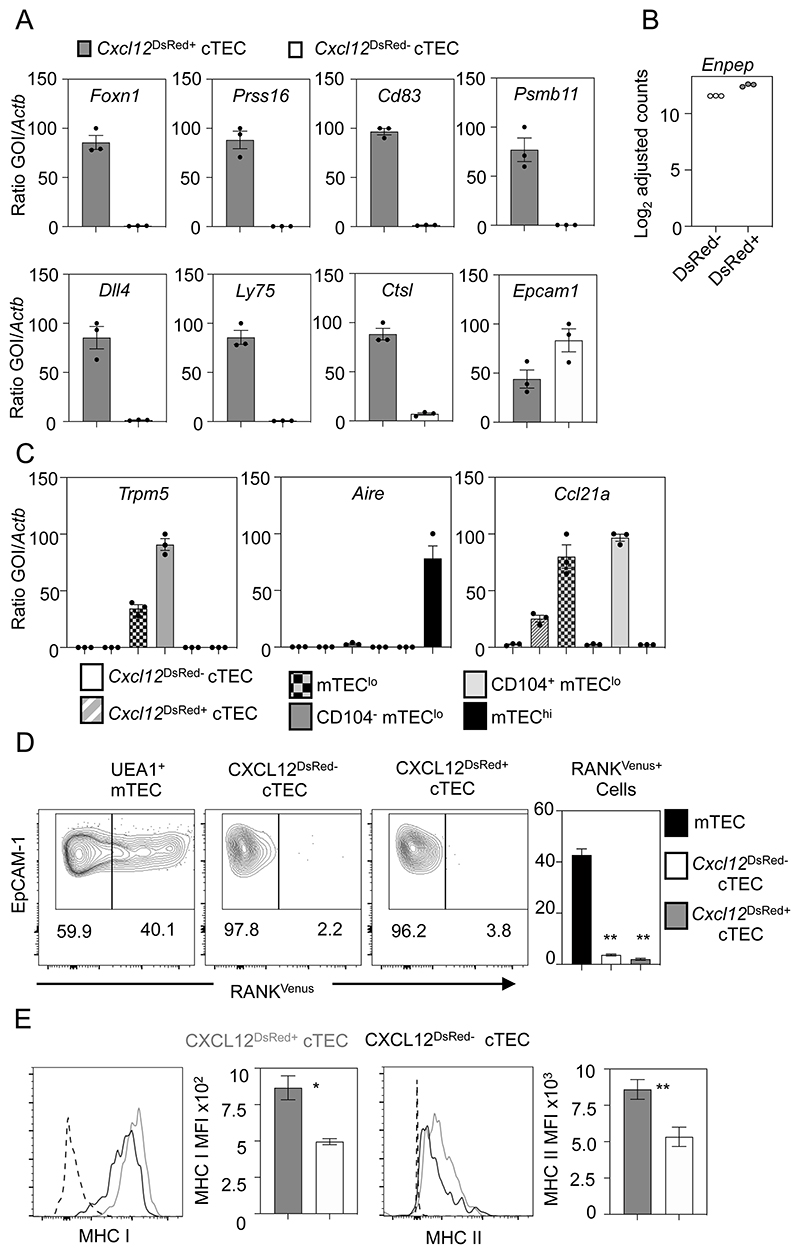
*Cxcl12^DsRed-^* cTEC Lack Expression of Foxn1 and A FOXN1 Target Gene Signature. (A) shows analysis of gene expression by qPCR in *Cxcl12*^DsRed+^ (gray bars) and *Cxcl12*^DsRed-^ (white bars) cTEC that were FACS-sorted from 10 week *Cxcl12^DsRed^* mice. (B) shows levels of expression of *Enpep* obtained from bulk RNA sequencing data in *Cxcl12*^DsRed+^ and *Cxcl12*^DsRed-^ cTEC. (C) shows qPCR analysis of mTEC-expressed genes *Trpm5*, *Aire* and *Ccl21a* in *Cxcl12*^DsRed+^ and *Cxcl12*^DsRed-^ cTEC compared to relevant mTEC subsets. For all qPCR, graphs represent of data obtained from at least 2 independentally sorted biological samples, with dots showing technical repeats. Error bars represent mean ± SEM (D) Flow cytometric analysis of RANK^Venus^ expression by total UEA1^+^ mTEC, *Cxcl12*^DsRed+^ and *Cxcl12*^DsRed-^ cTEC from Cxc12^DsRed^RANK^Venus^ reporter mice, n=5 from 5 separate experiments. (E) Flow cytometric analysis of indicated cell surface markers in *Cxcl12*^DsRed+^ (gray line) and *Cxcl12*^DsRed-^ (black line) cTEC from 10 week old Cxcl12^DsRed^ mice. Control staining levels obtained via omission of primary antibodies are shown as a grey line. Panel (E) also shows MFI analysis of indicated markers in *Cxcl12*^DsRed^ cTEC subsets. Data is from least 3 experiments, for MHC II n=8, and MHC I n=4. P values are as follows and indicate the significance relative to P1, using a Mann-Whitney non-parametric test: *, P < 0.05; **, P < 0.01; ***, P < 0.001; and ****, P < 0.0001. Error bars represent mean ± SEM.

To understand the events underlying this cTEC heterogeneity, we used RNA sequencing to compare the transcriptomes of *Cxcl12*^DsRed+^ and *Cxcl12*^DsRed-^ cTEC. Here, *Cxcl12*^DsRed+^ and *Cxcl12*^DsRed-^ subsets of total CD45^-^EpCAM1^+^UEA1^-^Ly51^+^ cTEC were FACS-sorted from 10 week old adult *Cxcl12*^DsRed^ reporter mice, with experiments performed in triplicate to produce 3 independent biological replicates for each subset. This approach identified 946 genes differentially expressed between DsRed^+^ and DsRed^-^ cTEC (Fig 2A). Much of this transcriptomic difference was driven by the lower expression of genes known to be direct targets of FOXN1 in *Cxcl12*^DsRed-^ cTEC relative to *Cxcl12*^DsRed+^ cTEC, and this correlated with the lack of expression of *Foxn1* in the former (p < 0.0001, Wilcoxon rank sum test; Fig 2B-D) (18). For example, the heatmap analysis in Figure 2D shows clear differences in expression of *Foxn1* and several of its direct targets including *Cxcl12*, *Dll4*, *Cd83*, *Ccl25*, *Ly75*, *Psmb11*, and *Prss16*. Further quantitative PCR (qPCR) analyses confirmed data obtained from RNAseq experiments, including the absence of *Foxn1* transcripts in *Cxcl12*^DsRed-^ cTEC (Fig 3A), as well as the absence of transcripts encoding FOXN1 target genes that play key roles in specific stages of thymocyte development including thymocyte migration (*Cxcl12*), Notch signalling (*Dll4*), and antigen processing/presentation (*Prss16*, *Psmb11*, *Ctsl*, *Ly75*). By contrast, *Cxcl12*^DsRed+^ and *Cxcl12*^DsRed-^ cTEC subsets showed no reduction in levels of *Epcam1* mRNA (Fig 3A). Importantly, *Cxcl12*^DsRed+^ and *Cxcl12*^DsRed-^ cTEC showed comparable levels of *Enpep* expression, the gene encoding the cTEC marker Ly51 (Figure 3B). qPCR analysis showed both *Cxcl12*^DsRed+^ and *Cxcl12*^DsRed-^ cTEC subsets lacked expression of mTEC markers, including the tuft cell marker *Trpm5*, as well as *Aire* and *Ccl21a* that were readily detectable within mTEC subsets (Figure 3C). Moreover, by crossing *Cxcl12*^DsRed^ with RANK^Venus^ reporter mice, we saw both cTEC subsets lacked expression of RANK, a key marker and regulator of mTEC (Figure 3D). These findings support the idea that *Cxcl12*^DsRed-^ Ly51^+^UEA1^-^ cells belong to the cTEC lineage, and do not contain mTEC lineage cells. Finally, although both *Cxcl12*^DsRed+^ and *Cxcl12*^DsRed-^ cTEC expressed MHC class I and MHC class II, their cell surface expression levels were significantly lower on *Cxcl12*^DsRed-^ cTEC (Fig. 3E).

**Figure 4:**
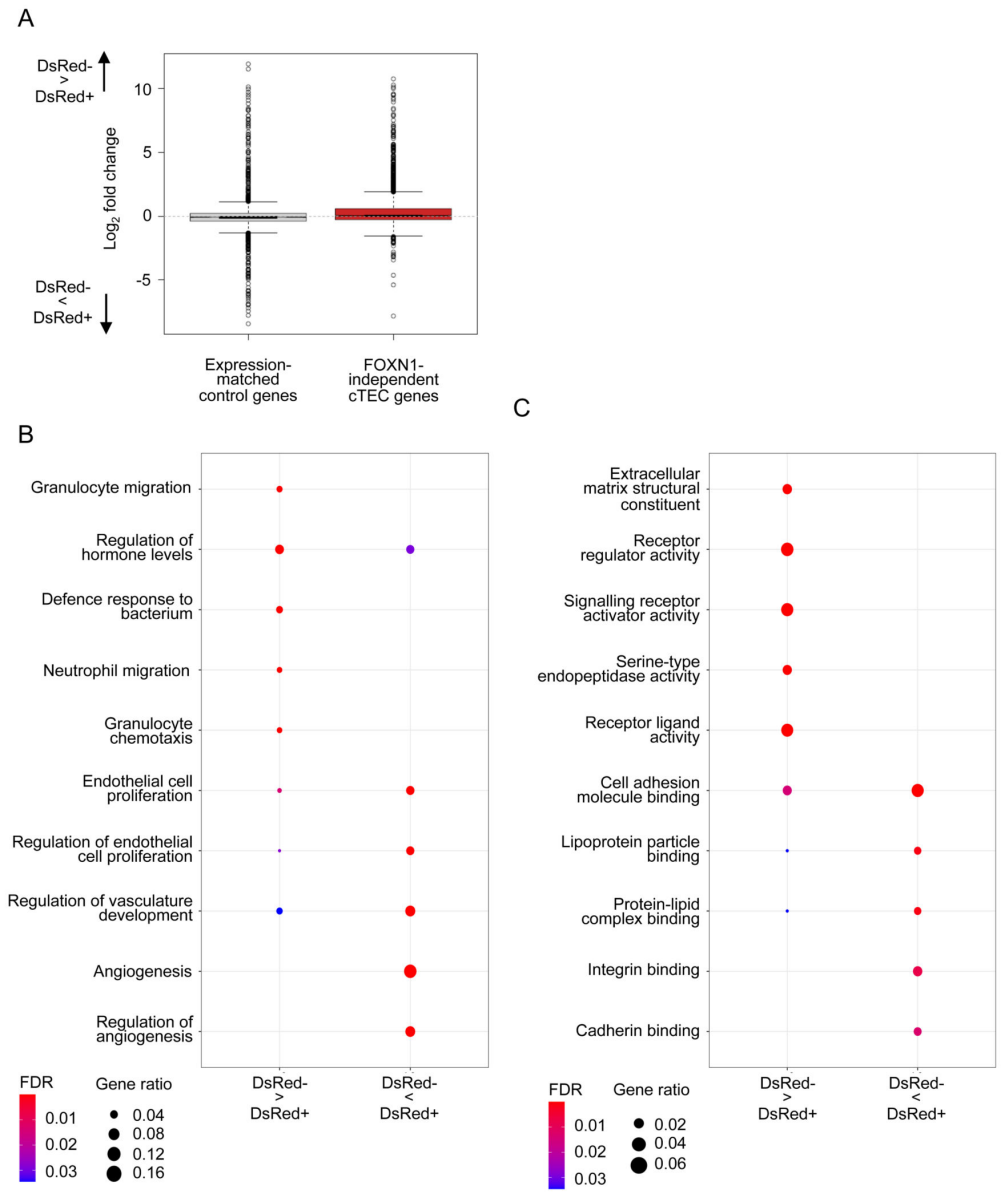
Comparative Analysis of Gene Expression In *Cxcl12*^DsRed+^ and *Cxcl12^DsRed-^* cTEC. (A) shows a boxplot of Foxn1-independent cTEC gene expression and genes matched by decile expression. cTEC marker genes were defined as those expressed more highly in perinatal or mature cTEC than other cell types in a reference dataset (36). cTEC markers that were Foxn1-enhanced (significantly upregulated or ≥0.25 log2 fold higher with increased Foxn1 (18) were removed to leave only FOXN1-independent cTEC markers. Expression of FOXN1-independent cTEC markers were similar between Cxcl12^DsRed+^ and Cxcl12^DsRed-^ cTEC. Below are shown dotplots of gene ontology analysis for biological processes (B) and molecular functions (C).

To examine further the nature of *Cxcl12*^DsRed-^ cTEC in relation to their *Cxcl12*^DsRed+^ counterparts, we searched for genes that were differentially expressed between the two subsets (Supp. Table 1). When we analysed the expression of cTEC marker genes (36), removing those known to be FOXN1-dependent (18), we saw the expression of cTEC marker genes in *Cxcl12*^DsRed+^ and *Cxcl12*^DsRed-^ cTEC subsets were similar (Figure 4A). Interestingly however, gene ontology analysis pointed towards some potential differences. For example, in *Cxcl12*^DsRed+^ cTEC we saw enrichment of pathways associated with regulation of endothelial cell proliferation, angiogenesis and vascular development, while *Cxcl12*^DsRed-^ cTEC showed enrichment of other distinct pathways including and serine-type endopeptidase activity regulation of granulocyte migration (Figure 4B, C). Collectively, these data suggest that while the major difference between *Cxcl12*^DsRed+^ and *Cxcl12*^DsRed-^ cTEC relates to expression of *Foxn1* and a FOXN1-dependent cTEC signature, they may also harbour gene expression patterns that point towards functional differences between the two subsets.

**Figure 5:**
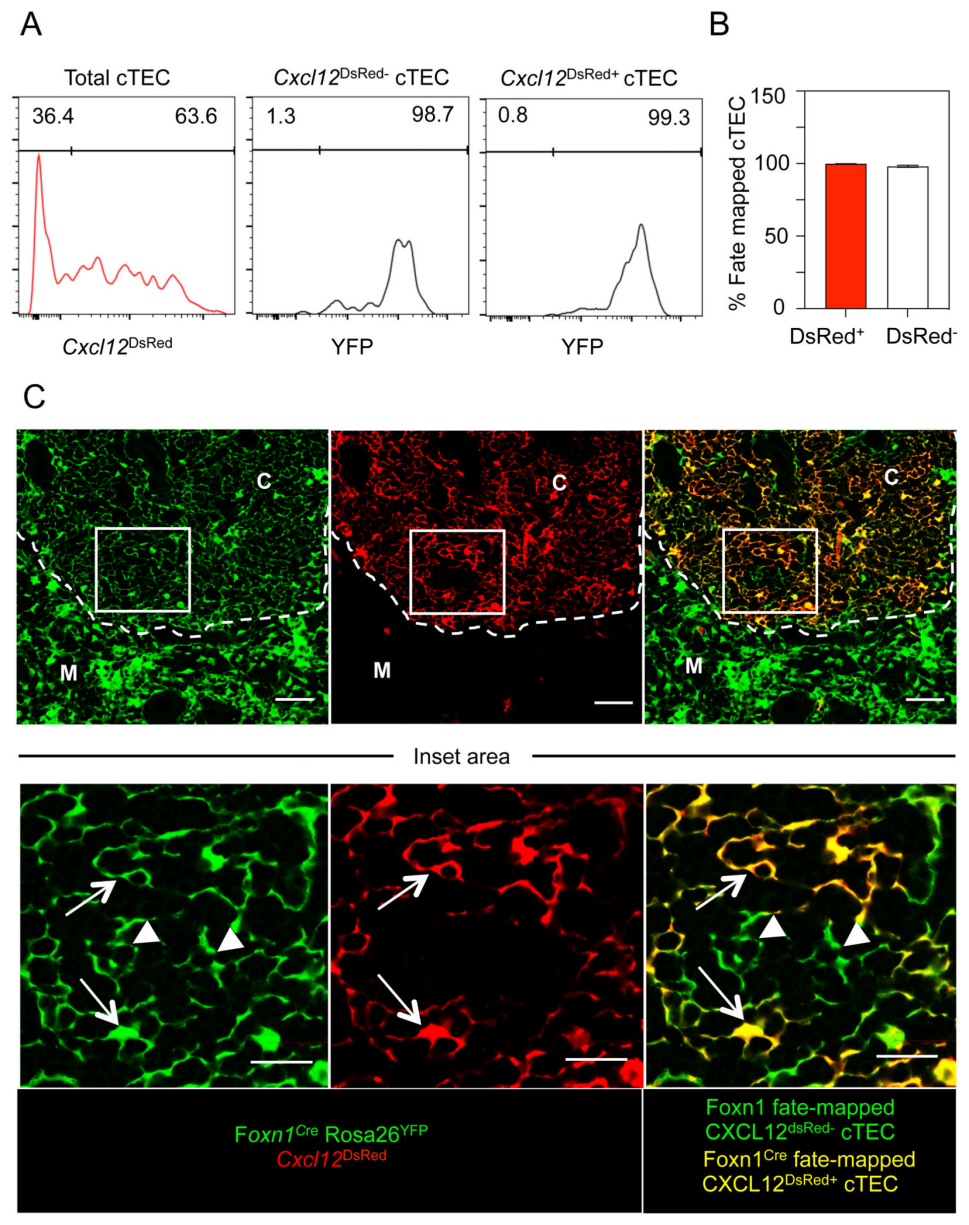
Both *Cxcl12*^DsRed+^ and *Cxcl12^DsRed-^* cTEC Are Derived From Foxn1-Expressing Cells. Panel (A) shows gating for the identification of *Cxcl12*^*DsRed*+^ and *Cxcl12*^*DsRed*-^ cTEC in 10 week old *Foxn1*^Cre^/Rosa26-YFP/*Cxcl12*^DsRed^ mice, and levels of YFP expression in these cells, where YFP indicates a history of Foxn1 expression. Gates are set following gating on YFP levels in CD45^+^ cells, where *Foxn1*^Cre^-mediated fate mapping is absent. Quantitation is shown in (B). Data is from 5 mice across 3 experiments. (C) shows confocal analysis of PFA-treated thymus sections from *Foxn1*^Cre^/Rosa26RYFP/*Cxcl12^DsRed^* mice, analyzed for expression of YFP (shown in green), DsRed (red), with co-expression appearing yellow. Upper panels are x10 magnification and show cortex (C) and medulla (M) areas defined by DAPI, dotted line is the CMJ. Scale bar denotes 50μm. The boxed area highlighted in upper panels represents an area of the cortex that is shown at x40 magnification in the image row below. Scale bar in the lower images denotes 20μm. Arrows identify *Foxn1*Cre-fate mapped YFP^+^*Cxcl12*^*DsRed*+^ cTEC, while arrowheads identify *Foxn1*Cre-fate mapped YFP^+^*Cxcl12*^*DsRed*-^ cTEC. Images are examples of 4 sections randomly chosen from 4 separate mice across 2 separate experiments.

The presence of *Foxn1*^-^ cTEC in the adult thymus could occur as a result of the downregulation of FOXN1 in cells that had previously expressed FOXN1, or via the progressive emergence of a cTEC subset with no prior history of FOXN1 expression. To distinguish between these possibilities, we used a fate mapping approach to examine the history of FOXN1 expression in *Cxcl12*^DsRed+^ and *Cxcl12*^DsRed-^ cTEC. In adult *Foxn1*^Cre^/*Rosa26*^YFP^/*Cxcl12*^DsRed^ mice, the vast majority of both *Cxcl12*^DsRed+^ and *Cxcl12*^DsRed-^ cTEC were *Foxn1*^Cre^ fate mapped (Fig. 5A, B), indicating both cTEC subpopulations were generated from *FOXN1* expressing cells. Confocal analysis of thymus sections from these mice demonstrated that both *Cxcl12*^DsRed+^ and *Cxcl12*^DsRed-^ *Foxn1^Cre^*-fate mapped cells were present within thymic cortex areas (Fig 5C). Use of confocal microscopy to further examine the phenotypic properties of cortex-resident *Cxcl12*^DsRed-^ cells was unfortunately hampered by the impact of PFA fixation, required to preserve DsRed protein, on successful antibody staining. Collectively, these findings show *FOXN1* is not uniformly expressed within the adult cTEC compartment, with the presence of *FOXN1^-^* cTEC providing an explanation for the presence of those cells that lack expression of the target gene *Cxcl12*. Importantly, our findings also show that heterogeneity in FOXN1 expression by cTEC extends beyond differences in *Cxcl12* expression and includes the differential expression of FOXN11-controlled loci (e.g. *Dll4*, *Ccl25*, *Psmbl1*, *Prss16*) that are important in the regulation of cortical T-cell development. Despite this change in the cTEC-specific mRNA signature, *Cxcl12*^DsRed-^ cTEC continue to reside within cortical areas alongside their *Cxcl12*^DsRed+^ counterparts, where they contribute to the reticular epithelial network of the adult thymic cortex.

### Stage-Specific Thymocyte Crosstalk Regulates cTEC Heterogeneity

**Figure 6:**
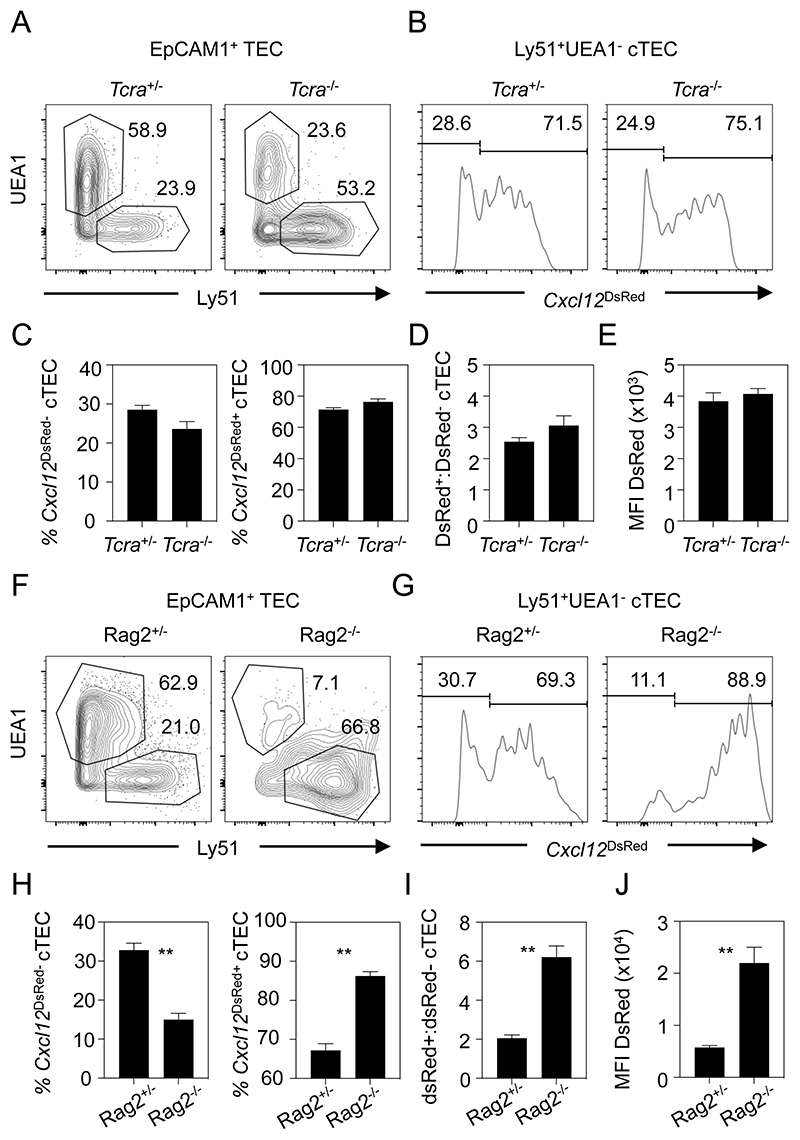
Stage-Specific Thymocyte Crosstalk Controls cTEC Heterogeneity. (A) shows identification of cTEC and mTEC in 10 week old *Cxcl12^DsRed^*/*Tcra*^-/-^ mice and *Cxcl12^DsRed^*/*Tcra*^+/-^ littermate controls, with panel (B) showing levels of *Cxcl12*^DsRed^ expression after gating on cTEC. Percentages (C) and ratios (D) of *Cxcl12*^DsRed+^ and *Cxcl12*^DsRed-^ cTEC in *Tcra*^-/-^ mice and *Tcra*^+/-^ mice are shown alongside MFI of DsRed in cTEC subsets (E). Panels (F-J) shows similar analysis of *Cxcl12*^DsRed^/*Rag2*^-/-^ mice and *Cxcl12*^DsRed^/*Rag2*^+/-^ littermate controls. All data are representative of at least 3 independent experiments, using the following numbers of mice: *Cxcl12*^DsRed^/*Tcra*^-/-^ n=12; *Cxcl12*^DsRed^/*Tcra*^+/-^ n=10; *Cxcl12*^DsRed^/*Rag2*^-/-^ n=6; *Cxcl12*^DsRed^/*Rag*^+/-^ n=6. P values indicate significance using a Mann-Whitney non-parametric test: **, P < 0.01. Error bars represent mean ± SEM.

Signals from developing thymocytes are known to regulate the development and formation thymic microenvironments, a process termed thymic crosstalk (37, 38). Much of our understanding of this process comes from studies examining the cellular interactions that govern events in the thymus medulla. For example, crosstalk with mTEC regulates development of Aire^+^ mTEC (39, 40) and post-Aire stages (29, 41). In contrast, how thymic crosstalk signals influence the thymic cortex, and in particular how they might control the Cxcl12/Foxn1 cTEC heterogeneity described here, is unclear. To examine this specific aspect, we crossed *Cxcl12*^DsRed^ mice with *Rag2^-/-^* and *Tcra^-/-^* mice, where T-cell development is blocked at the CD4^-^CD8^-^ or CD4^+^CD8^+^ stages, respectively. Interestingly, in *Tcra^-/-^ Cxcl12*^DsRed^ mice, cTEC heterogeneity was comparable to littermate controls (Fig 6A,B), with no alterations in the proportions of *Cxcl12*^DsRed+^ and *Cxcl12*^DsRed-^ cTEC (Fig. 6C) or the ratio of DsRed^+^:DsRed^-^ cTEC (Fig. 6D). MFI levels of DsRed in *Cxcl12*^DsRed+^ cTEC were also comparable (Fig. 6E). Thus, the appearance of *Cxcl12*^DsRed-^ cTEC occurs normally in the absence of CD4^+^ and CD8^+^ single positive thymocytes, suggesting that positive selection of CD4^+^CD8^+^ thymocytes is not essential for the generation of *Cxcl12*^DsRed^ cTEC heterogeneity. In contrast, when we performed similar analysis of *Rag2^-/-^Cxcl12*^DsRed^ mice (Fig. 6F), we saw that the proportion of *Cxcl12*^DsRed-^cTEC was decreased, with a concomitant increase in *Cxcl12*^DsRed+^ cTEC (Fig. 6G, H). This finding was accompanied by a skewing of the DsRed^+^:DsRed^-^ cTEC ratio in favour of DsRed^+^ cells (Fig 6I), with *Cxcl12*^DsRed+^ cTEC in *Rag2*^-/-^ mice also showing higher levels of DsRed compared to littermate controls (Fig 6J). These findings show that in the absence of CD4^+^CD8^+^ thymocytes, the appearance of *Cxcl12*^DsRed-^ cTEC is impaired, suggesting that maturation of CD4^-^CD8^-^ thymocytes is an important regulator of cTEC heterogeneity in the adult thymus.

**Figure 7:**
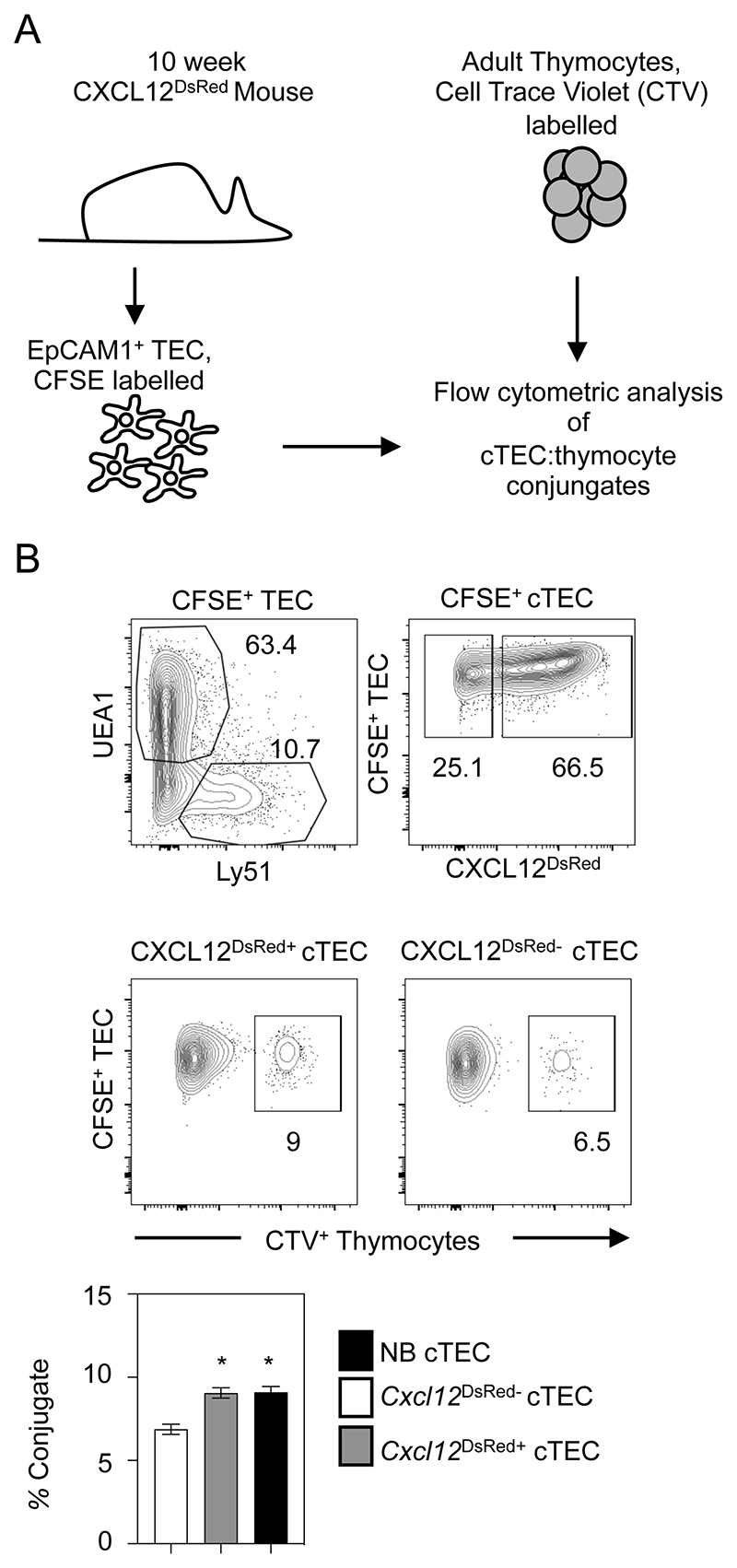
*Cxcl12^DsRed-^* cTEC Demonstrate An Impaired Capacity For Thymocyte Interactions. Panel (A) shows the experimental approach used to study cTEC:thymocyte conjugate interactions using flow cytometry. Panel (B) shows the gating approach used to compare the ability of *Cxcl12*^*DsRed*+^ and *Cxcl12*^*DsRed*-^ cTEC to form conjugates with thymocytes. Successful thymocyte/cTEC conjugates appear as CFSE^+^TEC:CTV^+^ thymocyte events within *Cxcl12DsRed*^+^ and *Cxcl12DsRed*^-^ cTEC subsets. Quantitation of cTEC:thymocyte conjugate formation is also shown in (B), with comparison of conjugate formation with *Cxcl12DsRed*^+^ cTEC (gray bar), *Cxcl12^DsRed-^* (white bar) cTEC, and neonatal cTEC (black bar). All data is representative of 4 individual experiments and 4 samples. P values indicate significance using a Mann-Whitney non-parametric test: *, P < 0.05. Error bars represent mean ± SEM.

The functional ability of cTEC is regulated by their expression of several key genes now known to be Foxn1 targets (18). Interestingly, a recent study (42) has shown that the formation of successful cellular interactions with thymocytes requires CXCL12 and DLL4, both of which are Foxn1 targets that are absent from *Cxcl12*^DsRed-^ cTEC. Given these differences between *Cxcl12*^DsRed+^ and *Cxcl12*^DsRed-^ cTEC, we wondered whether this may have functional consequences for their abilities to influence T-cell development. To investigate this, we performed a flow cytometry based cell conjugate assay where TEC-thymocyte interactions occur in a TCR-MHC independent manner (35) to compare the ability of *Cxcl12*^DsRed+^ and *Cxcl12*^DsRed-^ cTEC to form successful TEC-thymocyte conjugates. Here, purified EpCAM1^+^ TEC were FACS-sorted from adult *Cxcl12*^DsRed^ mice, labelled with the fluorescent dye CFSE, and mixed with Cell Trace Violet labelled thymocytes at a ratio of 5:1 Thymocytes:TEC (Fig 7A). Following centrifugation and 20 minutes incubation, pellets were gently disrupted and conjugate formation was assessed by flow cytometry following gating on *Cxcl12*^DsRed+^ and *Cxcl12*^DsRed-^ cTEC within the total cTEC population (Fig 7B). While both *Cxcl12*^DsRed+^ and *Cxcl12*^DsRed-^ cTEC were capable of conjugate formation, we saw a significant decrease in conjugates formed from *Cxcl12*^DsRed-^ cTEC (Fig 7B), suggesting *Cxcl12*^DsRed-^ cTEC may be less effective than their *Cxcl12*^DsRed+^ counterparts to influence T-cell development. Interestingly, when we compared the efficiency of TEC-conjugate formation using adult *Cxcl12*^DsRed+^ cTEC and neonatal cTEC, the latter being uniformly *Cxcl12*^DsRed+^ (Fig. 1C), we found them to be equally effective in mediating thymocyte interactions (Fig. 7B). Thus, the ability of *Cxcl12*^DsRed+^ cTEC to influence cortex-dependent thymocyte development may be consistent throughout the life-course, and any changes in this process may occur as a result of the progressive emergence of *Cxcl12*^DsRed-^ cTEC.

## Discussion

Interactions between thymocytes and cTEC/mTEC populations support the intrathymic development and selection of αβT-cells. Through examination of the cTEC compartment, we identified a developmentally regulated programme of heterogeneity which occurs over the life course and is defined by loss of expression of Foxn1 and its downstream targets. While our finding that all TEC arise from Foxn1-expressing cells is consistent with previous reports (20), what causes some cTEC to downregulate Foxn1, and Foxn1 dependent genes, is not known. Importantly, while Foxn1^-^ TEC have been described previously (43–45), multiple features including their intrathymic positioning, transcriptomic profile, and intrathymic generation have remained poorly understood. Here, by identifying the gene profile of these cells, including their loss of a functionally important cTEC gene signature, we provide evidence they are transcriptionally distinct from their Foxn1-expressing counterparts. Moreover, the intrathymic positioning within the cortex of the cTEC subsets defined here, together with their regulation by CD4^-^CD8^-^ but not CD4^+^CD8^+^ thymocytes, extends our understanding of complexity of the cTEC compartment and the mechanisms that control this. Indeed, as the appearance of cTEC that lack Foxn1 and its key target genes is regulated by thymocyte crosstalk, in particular events specific to CD4^-^CD8^-^ thymocytes, it may be that early stages of T-cell development generate signals that cause loss of Foxn1, which then results in cTEC heterogeneity. Interestingly, analysis from birth up to 20 weeks of age showed that the frequency of *Cxcl12*^DsRed-^ cTEC had plateaued by around 10 weeks, which may indicate that turnover of *Cxcl12*^DsRed-^ cells takes place, rather than a process that results in their progressive accumulation during the life course.

The presence within the adult thymic cortex of cTEC that no longer express key genes regulating specific stages of thymocyte development raises multiple interesting scenarios. For example, it may be relevant to understanding progressive changes in thymus function under homeostatic conditions. Here, as both *Cxcl12* and *Dll4* are important regulators of the β-selection checkpoint. (46), absence of these genes in *Foxn1*^-^ cTEC may impact the ability of the thymus to support transition to the CD4^+^CD8^+^ stage. Also significant is that while *Psmb11*, the cTEC-specific gene encoding the thymoproteosome component β5t, is unique to cTEC (12), our data suggest that not all adult cTEC express transcripts of *Psmb11*. Thus, it may be the case that in the adult thymus, both *Psmb11*^+^ and *Psmb11*^-^ cTEC contribute to CD8^+^ SP positive selection, but they generate distinct αβTCR repertoires as a result of differences in the MHC class I bound self-peptides they can produce (thymoproteosome/β5t dependent peptides for *Cxcl12*^DsRed+^ cTEC versus non-thymoproteosome/β5t independent peptides for *Cxcl12*^DsRed-^ cTEC). Here, it is important to note that β5t-deficient mice are still able to positively select some SP8^+^ thymocytes (47), a finding that may be consistent with the scenario that cTEC lacking *Psmb11* can to contribute to SP8 generation in normal mice. Alternatively, adult *Foxn1*^-^ cTEC that lack *Psmb11* may be incapable of positive selection due to other functional defects, such as a failure to interact with CD4^+^CD8^+^ thymocytes. While it is interesting to note that *Cxcl12*^DsRed-^ cTEC express significantly lower levels of MHC class I relative to their *Cxcl12*^DsRed+^ counterparts, and form fewer cell-cell conjugates with thymocytes, further studies are required to examine the functional properties of the cTEC subsets described here. Relevant to this, our attempts to compare the functional abilities of FACS-sorted *Cxcl12*^DsRed+^ and *Cxcl12*^DsRed-^ cTEC from adult mice in reaggregate thymus organ cultures were unsuccessful. Here, intact 3-dimensional structures consistently failed to form when using TEC isolated from adult mice, which is in contrast to the efficient generation of intact RTOC from embryonic TEC (48, 49). The reasons for the inability of adult TEC to effectively form RTOC under conditions that support embryonic TEC reaggregation are not clear. However, it is interesting to note that early studies on the capacity of embryonic tissues to undergo effective reaggregation attributed this to their ability to undergo what was termed ‘inductive interactions’(50), which may be missing from adult TEC. Whatever the case, further studies are required to compare the functional capacity of cTEC subsets described here, which would also benefit from the creation of improved experimental systems to study adult TEC functions in vitro.

Beyond directly influencing specific stages of thymocyte development, *Cxcl12*^DsRed-^ cTEC may also play role in physically supporting the epithelial scaffold within the thymus cortex, a possibility raised recently in the context of the presence of FOXN1^-^ TEC in thymus (44). Such a possibility may be compatible with our finding that *Cxcl12*^DsRed-^ cTEC are interspersed in the cortex alongside *Cxcl12*^DsRed+^ cells. A final possibility is that alongside loss of Foxn1-mediated functional properties, *Cxcl12*^DsRed-^ cTEC acquire new functional features that are important in adult thymus cortex organisation and/or function. Again, further examination requires approaches to directly assess the functional properties of defined cTEC subsets.

In sum, we show that the Ly51^+^UEA1^-^ cTEC compartment undergoes developmentally regulated changes in its cellular makeup that are driven by interactions with the maturation of immature CD4^-^CD8^-^ thymocytes. We identify the emergence of a cTEC subset that retains its Ly51^+^UEA1^-^ phenotype and positioning within the cortex, but has ceased to express Foxn1, resulting in the lack of expression of key Foxn1 target genes that define the functional properties of cTEC. These findings demonstrate the emerging complexity of the thymic cortex, and will aid in future studies that examine the role of this intrathymic site in thymocyte development.

## Supplementary Material

Supplementary Table 1

## Key Points

1. cTEC undergo progressive changes during postnatal life caused by loss of *Foxn1*.
2. *Foxn1*^-^ cTEC remain within the cortex, but lack cTEC signature genes.
3. *Foxn1*^-^ cTEC show an impaired ability to influence developing thymocytes.
